# 3D printed personalized titanium plates improve clinical outcome in microwave ablation of bone tumors around the knee

**DOI:** 10.1038/s41598-017-07243-3

**Published:** 2017-08-08

**Authors:** Limin Ma, Ye Zhou, Ye Zhu, Zefeng Lin, Lingling Chen, Yu Zhang, Hong Xia, Chuanbin Mao

**Affiliations:** 1 Department of Orthopedics, Guangdong Key Lab of Orthopedic Technology and Implant, Guangzhou General Hospital of Guangzhou Military Command, 111 Liuhua Road, Guangzhou, 510010 China; 2 Department of Chemistry and Biochemistry, Stephenson Life Sciences Research Center, University of Oklahoma, Norman, OK 73019 USA; 3 School of Materials Science and Engineering, Zhejiang University, Hangzhou, Zhejiang, 310027 China

## Abstract

Microwave ablation has been widely accepted in treating bone tumor. However, its procedure is time-consuming and usually results in postoperative fractures. To solve this problem, we designed and fabricated titanium plates customized to the patients’ bone structures. The personalized titanium plates were then used for fixation after the removal of tumorous tissue. Specifically, 3D models of tumor-bearing bone segments were constructed by using computed tomography (CT) and magnetic resonance imaging (MRI). The 3D models were used to design the personalized titanium plates. The plate model was transferred into a numerical control machine for manufacturing the personalized titanium plates by 3D printing. The plates were then surgically implanted for reconstruction assistance following microwave-induced hyperthermia to remove the bone tumor. Implementation parameters and knee functions were then evaluated. No postoperative fractures, implant failures or loosening problems occurred; mean Musculoskeletal Tumor Society score was 27.17 from the latest follow-up. Mean maximum flexion of affected knees was 114.08°. The results of knee gait analysis were comparable with normal population data. Our work suggests that personalized titanium plates can significantly improve the clinical outcomes in the surgical removal of bone tumor. This study represents the first-time effort in using personalized titanium plates for such surgery.

## Introduction

Due to the progress made in chemotherapy, imaging technology and biomaterials over the past few decades, limb-salvage surgery has become a standard procedure for most bone tumor and has obtained numerous encouraging outcomes. Various approaches, such as endoprosthetic replacement, allograft transplantation, or microwave ablation, have been used for reconstruction of limbs after tumor resections^1–5^. Microwave ablation has received widespread acceptance in the treatment of various bone and soft tissue tumor^4–9^. For example, Fan *et al*.^4, 5^ reported the use of microwave ablation to remove malignant bone tumor of the extremities and pelvis. They found that the microwave ablation could improve the function of the salvaged limbs and reduce complication rates^4, 5^.

Traditionally, bone defect was generated after microwave ablation and surgical removal of bone tumor. However, such defect may induce a relatively high postoperative fracture rate in the early stages^4^. For instance, Fan *et al*.^4, 10^ revealed that 7% of alive patients suffered from postoperative fractures and hence revision or amputation was needed. They showed that prophylactic fixation, grafting, and partial weight bearing could reduce the fracture rate to some extent. In the cases of limb tumors treated with other methods such as irradiation and autoclave^11–14^, the fracture rate ranged from 3.7% to 29%. Hence postoperative fracture was a major complication of reconstruction after diaphyseal tumor resection.

The fracture happened in spite of the bone cavities being filled with a mixture of bone graft and cement and a prophylactic fixation procedure being performed. Therefore, a more rigid internal fixation may be needed to avoid postoperative fractures when an implant is used to fill the bone cavities. The traditional fixation is guided by means of a non-personalized titanium plate. However, the morphological change of the bone involved in the resection of a bone tumor and hyperthermia treatment is liable to cause misalignment between the anatomic plate (usually employed for traumatic fractures) and the bone surface. In addition, the current non-personalized titanium plate used for internal fixation makes the bone tumor removal time-consuming. Hence, optimizing the plate system used for fixing the bone after microwave ablation is necessary to improve the effectiveness of bone reconstruction. Recently, toward this goal, the utility and encouraging clinical outcomes of computer-aided design (CAD)/computer-aided manufacture (CAM) techniques in the orthopedic fields have been reported^15–18^. For example, Xu *et al*.^17^ demonstrated the low risk involved in intra-articular penetration and the low rate of implant failure when applying a custom-made plate to assist the repairing of acetabular fractures.

**Figure 1:**
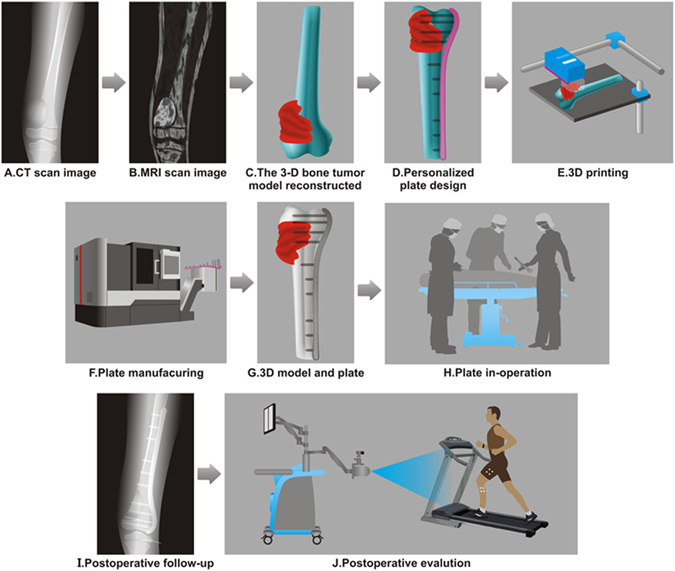
The current global procedure for the designed clinical study. (**A**) CT scan is first performed on the tumorous bone. (**B**) The MRI scan image is then performed on the tumorous bone. (**C**) In accordance with the merged CT-MR image, a 3D model of the tumorous bone is constructed using Mimics software, with tumor being highlighted in red. (**D**) The 3D model shown in C is then used to design a personalized titanium plate (pink) using UG software. (**E**) 3D printing is used to print the bone model shown in (**C**). (**F**) A numerical control machine is used to fabricate the personalized titanium plate shown in (**D**). (**G**) The fabricated titanium plate is combined with the 3D printed tumorous bone to match the model shown in (**D**) in order to test if the plate matches the tumorous bone as modeled. (**H**) The fabricated personalized plate is then used to fix the bone structure after the surgery removal of osteosarcoma and the bone repair using the mixture of allograft bone and cement. (**I**) The recovery of the patient is followed by X-ray examination and gait analysis.

To reduce the postoperative fracture rate of internal fixation following microwave ablation and improve the efficiency of reconstruction, in the current study, we used CAD/CAM techniques to design and manufacture personalized (i.e. customized) plates to assist with bone reconstruction following microwave ablation of bone tumors. The general strategy of our study is schematically illustrated in Fig. 1. For the first time, we used the personalized titanium plates, which are made aligned well with the anatomical bone surface of the specific patient, to achieve excellent internal fixation after bone tumor resection.

## Materials and Methods

### Patients

Between November 2013 and December 2015, twelve patients (Table 1) with bone tumors in the distal femur or proximal tibia were treated surgically with microwave-induced hyperthermia *in situ* and reconstruction with personalized titanium plates customized to each patient. These patients do not have metastatic tumors. This study was conducted according to the protocol approved by the Institutional Review Board of Guangzhou General Hospital of Guangzhou Military Command. Informed consent was received from all patients regarding both participation in this study and publishing of the results of this study.

**Table 1:** The demographic characteristics of 12 patients (Inclusion criteria: Clinical diagnosis of primary malignant bone tumors in the distal femur or proximal tibia according to the preoperative biopsy; Bearing malignant soft tissue tumor that invades distal femur or proximal tibia.

Patient	Age	Sex	Diagnosis	Chemotherapy	Follow-up (month)/Status
1	23	Male	Giant cell tumor of the right distal femur	N	27/Alive
2	18	Male	Ewing’s sarcoma of the right distal femur	Y	25/Alive
3	8	Male	Osteosarcoma of the left distal femur	Y	16/Alive
4	17	Male	Osteosarcoma of the right distal femur	Y	23/Alive
5	54	Male	Giant cell tumor of the left distal femur	N	29/Alive
6	29	Female	Giant cell tumor of the left proximal tibia	N	33/Alive
7	18	Male	Osteosarcoma of the right proximal tibia	Y	34/Alive
8	15	Male	Osteosarcoma of the right proximal tibia	Y	Died of lung metastases after 14 months
9	26	Male	Chondrosarcoma of the left distal femur	Y	33/Alive
10	19	Male	Osteosarcoma of the left distal femur	Y	36/Alive
11	23	Male	Osteosarcoma of the right proximal tibia	Y	42/Alive
12	24	Female	Osteosarcoma of the right distal femur	Y	40/Alive

Exclusion Criteria: Distant metastasis; Benign tumor; Metastatic tumor around the knee; No indication of limb salvage; Malignant soft tissue tumor without bone invasion; Recrudescent tumors).

Ten males and two females with a mean age of 22.8 years (range, 8 to 54 years) were recruited for this study. There were 8 patients with tumor in the distal femur and 4 patients with tumor in the proximal tibia. Details concerning patient criteria are provided in Table 1. A preoperative biopsy was performed on every patient to verify his or her diagnosis. Patients with osteosarcoma or Ewing’s sarcoma received one to two courses of chemotherapy. Patients diagnosed with a giant cell tumor did not receive chemotherapy but were instructed to receive bisphosphonate treatment before surgery. Then examinations by 64-slice spiral computed tomography (CT, Siemens, Germany, Fig. 1A) and magnetic resonance imaging (MRI, MAGNETOM Trio, Siemens, Germany, Fig. 1B) were performed to re-assess tumor aggressiveness and obtain the DICOM (Digital Imaging and Communications in Medicine) data for subsequent preoperative simulations.

### Preoperative simulation

Bilateral femoral or tibial CT data with DICOM formatting was obtained and imported into Mimics 15.0 software (Belgium) for three-dimensional (3D) reconstruction of the bone. By this software, a 3D model of the tumor was reconstructed with the MRI image data. The model was then used to identify the range of the lesion, which is used to guide the ablation procedure and customized plate design (Fig. 1C). In accordance with the merged CT and MRI images, a rapid prototyping (RP) model of the affected bone segment was manufactured with a 3D printing system (MED610 purchased from Objet Ltd, Israel, Fig. 1E) in order to visualize the area of the lesion and define the surgical procedure (Fig. 1H).

### Plate design and manufacturing

**Figure 2:**
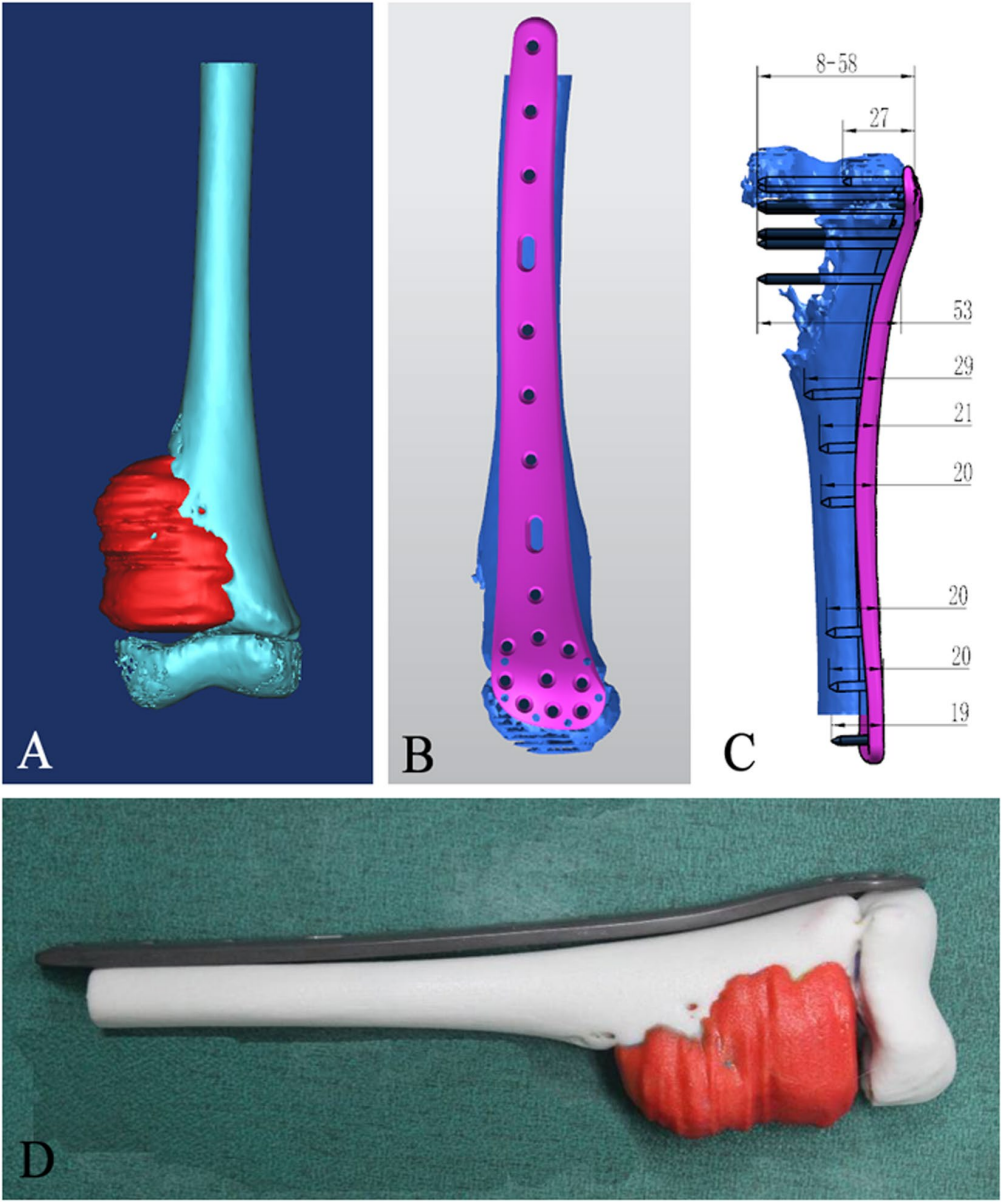
The modeling of the tumorous bone and personalized titanium plate as well as the matching between the tumorous bone and the titanium plate. (**A**) The 3D model of the tumorous bone was constructed with Mimics software according to the merged CT-MR data. (**B**) The 3D model of the tumorous bone is matched with a personalized titanium plate model (pink) using UG software. (**C**) The resultant 3D model is filled with screws in order to show the required number as well as the location and lengths of the screws that will be used during the operation. (**D**) The 3D printed tumorous bone model is matched with a fabricated personalized titanium plate.

The intact side of the tumor-bearing bone was determined to be a suitable attachment site for the plate (Fig. 2B). Since there was no adequate normal surface of bone in the area affected by the tumor for attachment, the contralateral femur or tibia was reconstructed into 3D images for plate design. The length of the plate was dependent on the range of ablation and the length of femur or tibia. More than 3 locking holes were designed at the proximal part of the plate. More than 6 locking holes and several additional holes with a diameter of about 1 mm were designed for the reconstruction of soft tissue (Fig. 1D). In order to theoretically improve the strength of the internal fixation system, the thickness of the plates (~6 mm) was larger than that of the traditional plates^19^ (~4.5 mm) used in repairing the proximal tibia or distal femur fractures. In the 3D view, the required number, location and length of screws were determined preoperatively following the simulated surgical procedure (Fig. 2C).

Afterwards, the plate data was saved in the IGES file format and input into Unigraphics NX version 9.0 software (UG, Siemens PLM Software, Co, Ltd) for programming. The generated code was then transferred to a computerized numerical control (CNC) digital milling machine (FANUC Co, Ltd, Yamanashi, Japan) to manufacture the plates with drilled holes. We chose Ti-6Al-4V ELI medical titanium (ASTM F136, Baoji Unique Titanium Industry Co, Ltd, Shanxi, China) to manufacture the plates (Fig. 1F). The plates were trimmed to remove excess metal, cleaned, polished, and anodized. The locking screws assembled with the plate were manufactured and used to test if they could be stably installed. Thereafter, we placed the plate system on a RP model of the affected bone segment to test whether the plates matched the bone surface (Fig. 2D).

Before clinical implementation, the biomechanical property of the plate was evaluated with a universal material testing machine (ZG-056, MTS Systems Corporation; Eden Prairie, MN) following an ISO standard protocol (YY/T0342–2002). Static four-point bending tests were performed on 5 specimens. The inner span was 20 mm and outer span was 40 mm. The bending strength, bending stiffness and the yield load were measured at a crosshead speed of 0.05 mm/s. The results of biomechanical tests revealed that the bending strength, bending stiffness and yield load were 56.8 ± 1.2 (mean ± standard deviation) Nm, 5106.8 ± 905.2 N/mm, and 6375.5 ± 193.1 N, respectively, suggesting the plates are qualified for clinical application^20^.

### Surgical procedure

Before sterilization, the RP model and plate system were used to familiarize the patients with the procedure one day prior to the surgery. There were three stages in each surgery^4^. First, an appropriate operative approach was chosen. The tumor was separated from the surrounding normal tissues and exposed for the subsequent ablation. The soft tissue of the knee joint without a tumor invasion was reserved for the subsequent bone reconstruction. Second, the surrounding normal tissue was protected from thermal damage, and microwave ablation of the tumor tissue *in situ* was performed under specific settings. Finally, the devitalized tissue was resected (Fig. 1I). Then the mixture of allograft bone and bone cement (poly(methyl methacrylate)) was used to fill the resultant defect, followed by the fixation of the personalized plate to strengthen the bone segment. Parameters including operation time and blood loss were recorded.

### Postoperative care

Every involved knee was immobilized with a protective brace after surgery. In general, the patients were asked to experience exercising affected limbs and weight bearing at 3 to 4 weeks after operation. The radiography was arranged first after 2 days, then after 1, 3, 6 and 12 months postoperatively, and annually thereafter for patients, to evaluate bone healing. Monthly telephone investigations were performed to follow the patients’ viabilities and also to determine the occurrence and timing of complications. The radiographs were used to evaluate bone healing and the occurrence of fractures or implant failures. Implant failures were defined as breaking of internal fixation or slipping of the implants from the original anatomical location. The occurrence of infection, characterized by persistently unhealed wounds and local discharging, were also evaluated during the out-patient follow-ups.

### Functional evaluation

During the follow-ups within the same timeframe as above, limb function was evaluated with the Musculoskeletal Tumor Society (MSTS) scores using an optical knee analysis system (Opti_Knee^®^, Innomotion Inc., Shanghai, China)^21, 22^ (Fig. 1J). The MSTS system is an approach used to assess patients’ pain, functional limitation, walking distance, use of support, emotional acceptance, and gait^22^. The range of motion (ROM) in six degrees of freedom (6DOF, angle: flexion-extension, internal-external rotation, adduction-abduction; translation: antero-posterior, proximo-distal, medio-lateral) during the gait and the maximum flexion of affected knees were calculated and compared with the data of Chinese normal knees^21^.

## Results

### General result

The mean age of all 12 patients (10 males and 2 females) was 22.8 years (8 to 54) (Table 1). There were 7 cases of osteosarcomas, 3 cases of giant cell tumors, 1 case of Ewing’s sarcoma and 1 case of chondrosarcoma in this study. The mean follow-up time of all patients was 29.3 (14 to 42) months. The results of the parameters for the follow-up are detailed in Table 2. Specifically, the mean operation time was 272 min (slightly shorter than the reported 5 hours)^15^; the mean volume of blood loss was 388 mL; the mean MSTS score was 27.13 according to their last follow-up; and the mean value of maximum flexion of affected knees was 114.08° (105° to 128°). The results of knee ROMs in 6DOF during the gait are presented in Table 3, which is comparable with the normative data of Chinese gaits^21^.

**Table 2:** The results of implementation parameters.

Patient	Age	Sex	blood loss (mL)	operation time (min)	MTST scores	maximum flexion (°)
1	23	Male	50	185	27	111
2	18	Male	300	200	28	121
3	8	Male	600	316	27	113
4	17	Male	500	350	26	105
5	54	Male	150	160	26	118
6	29	Female	200	195	27	124
7	18	Male	500	325	28	108
8	15	Male	400	297	27	109
9	26	Male	450	275	27	107
10	19	Male	400	310	27	110
11	23	Male	600	366	29	128
12	24	Female	500	288	27	115
Mean ± SD	22.8 ± 11.3		387.5 ± 1173.3	272.3 ± 69.6	27.2 ± 0.8	114.1 ± 7.3

MTST, Musculoskeletal Tumor Society. SD, standard deviation.

**Table 3:** The results of the patients’ knee ROMs in 6DOF during gait (mean ± standard deviation).

Subjects	VR/VL (°)	IR/ER (°)	F/E (°)	A/P (mm)	P/D (mm)	M/L (mm)
Current study	6.8 ± 2.9	11.1 ± 3.4	52.5 ± 10.9	14.0 ± 4.7	13.3 ± 5.2	8.9 ± 4.1
Normative data^16^	9.3	10.9	57.4	12.8	15.0	9.4

F/E, flexion/extension; IR/ER, internal/external femoral rotation; VR/VL, varus/valgus; A/P, anterior/posterior femoral translation; P/D, proximal/distal femoral translation ML, medial/lateral femoral translation.

### Complication

One patient had local recurrence at 13 months after surgery and died of lung metastases 3 weeks later. The remaining 11 patients were disease free. According to the radiographies (See Supplementary Materials), no fractures, implant failures, or loosening problems occurred among these patients during the follow-ups. One patient had superficial infection, which was cured with debridement and antibiotic therapy. One patient had a peroneal nerve injury resulting from the excision during the operation due to a partial nerve located inside of the surgical margin.

### Preoperative simulation

**Figure 3:**
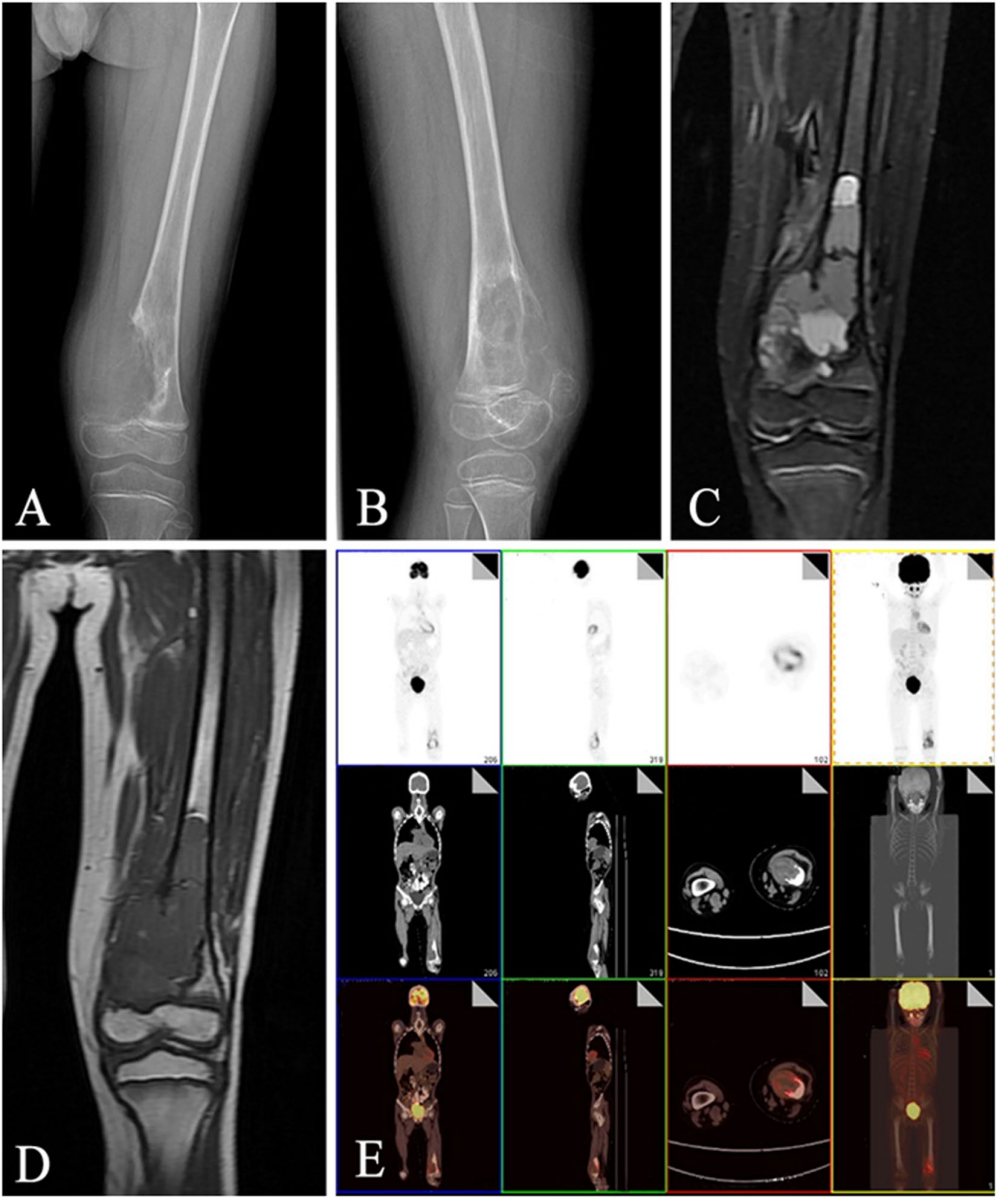
Preoperative imagological data. (**A**,**B**) Malignant tumor in the left distal femur was observed in anteroposterior (**A**) and lateral (B) X-ray radiographs. (**C**,**D**) Malignant tumor was confirmed from T1-weighted (**C**) and T2-weighted (**D**) magnetic resonance images. (**E**) Positron emission tomography-computed tomography was carried out to confirm the absence of metastasis.

In a specific case, an 8-year-old male was seen for left distal thigh pain and swelling for 1 month. Radiography provided imaging of an osteolytic lesion in the left distal femur and revealed a malignant bone tumor (Fig. 3A,B). Then MRI and an open biopsy were performed and confirmed the diagnosis of osteosarcoma in the left distal femur (Fig. 3C,D). Positron emission tomography-computed tomography (PET-CT) was carried out to classify the lesion as Enneking stage IIB (Fig. 3E). The patient received one course of preoperative chemotherapy (APMMI, adriamycin, paclitaxel, high-dose methotrexate, ifosfamide). Then he underwent examinations including MRI and CT. The MRI and CT images were further used for 3D reconstruction of left distal femur and postoperative evaluation in Mimics (Fig. 2A).

### Plate design and manufacturing

On the second day, the length of the plate was determined according to the range of tumor invasion observed in MRI (Fig. 2B). Five locking holes were designed at the proximal part of the plate and ten holes at the distal part. The orientation of screws was designed to avoid epiphyseal penetration, and the length of each screw needed was determined accordingly (Fig. 2C). On the third and fourth days, the six centimeter-thick plate, screws and a RP model of the tumor-bearing femur were manufactured to test the anatomic match of the plate preoperatively. The implants and the RP model were sterilized on the day before the surgery (Fig. 2D).

### Surgical procedure

**Figure 4:**
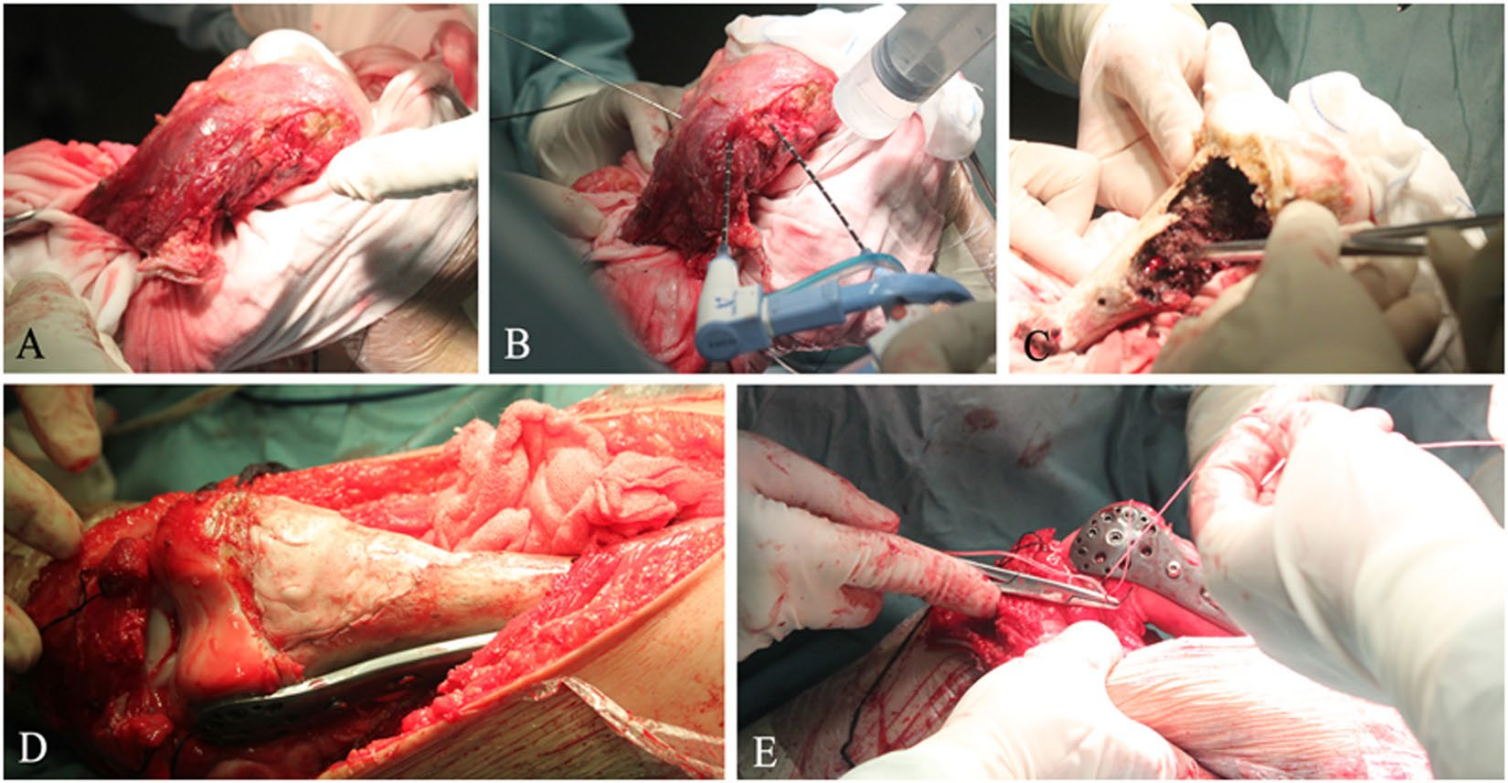
Surgical procedure. (**A**) Bone tumor was separated from the surrounding normal tissues with an adequate margin. (**B**) Microwave ablation of the tumor tissue was performed *in situ*. (**C**) The ablated tumor tissue was removed. (**D**) The resultant bone defect was filled with the mixture of allograft bone and cement, followed by the fixation with the personalized titanium plate. (**E**) The lateral collateral ligaments were sutured to the lateral epicondyle.

After induction of general anesthesia, a medial incision to access the distal thigh was made, including an excision of the biopsy site. The distal femur with vastus mesialis was exposed and isolated from the surrounding normal tissue (Fig. 4A). The patellar tendon and collateral ligaments were detached from their original attachment sites on the tibia and femoral condylar, respectively. Following the confirmation of the range of the lesion (including tumor invasion into the medullary cavity) under direct vision, the antennae, 2 cm apart, were implanted along the long axis of the femur. The normal tissues were protected from overheating by continuous injections of cold saline into a gauze placed between the tumor bone and the normal tissues (Fig. 4B).

According to the previous reports^4, 5^, the frequency output was set to 2450 MHz and the power was 100 W for microwave ablation in this study. A temperature-controlled system was chosen and two thermocouples were used to dynamically monitor the temperature of the tumor, the cortical bone, and the normal tissues during the surgery. The target temperature in the lesion area remained between 60 to 100 °C for 30 min, while the normal tissue temperature was kept lower than 39 °C. The devitalized tissue was removed after hyperthermia and the cavity was exposed (Fig. 4C). This cavity was then filled with the mixture of allograft bone and cement.

Before the cement was solidified, the customized plate was attached onto the bone surface, and screws were implanted based on the preoperative design (Fig. 4D). Actual observation during the surgery was also taken into account so extra screws were needed to enhance the stability and mechanical integrity of the mixture. The lateral collateral ligaments were sutured to lateral epicondyle through the customized smaller holes (Fig. 4E). The medial collateral ligament and patella tendon were anchored onto their intrinsic attachment sites. After the placement of a drain and wound closure, the knee joint was immobilized with a brace.

### Postoperative evaluation

**Figure 5:**
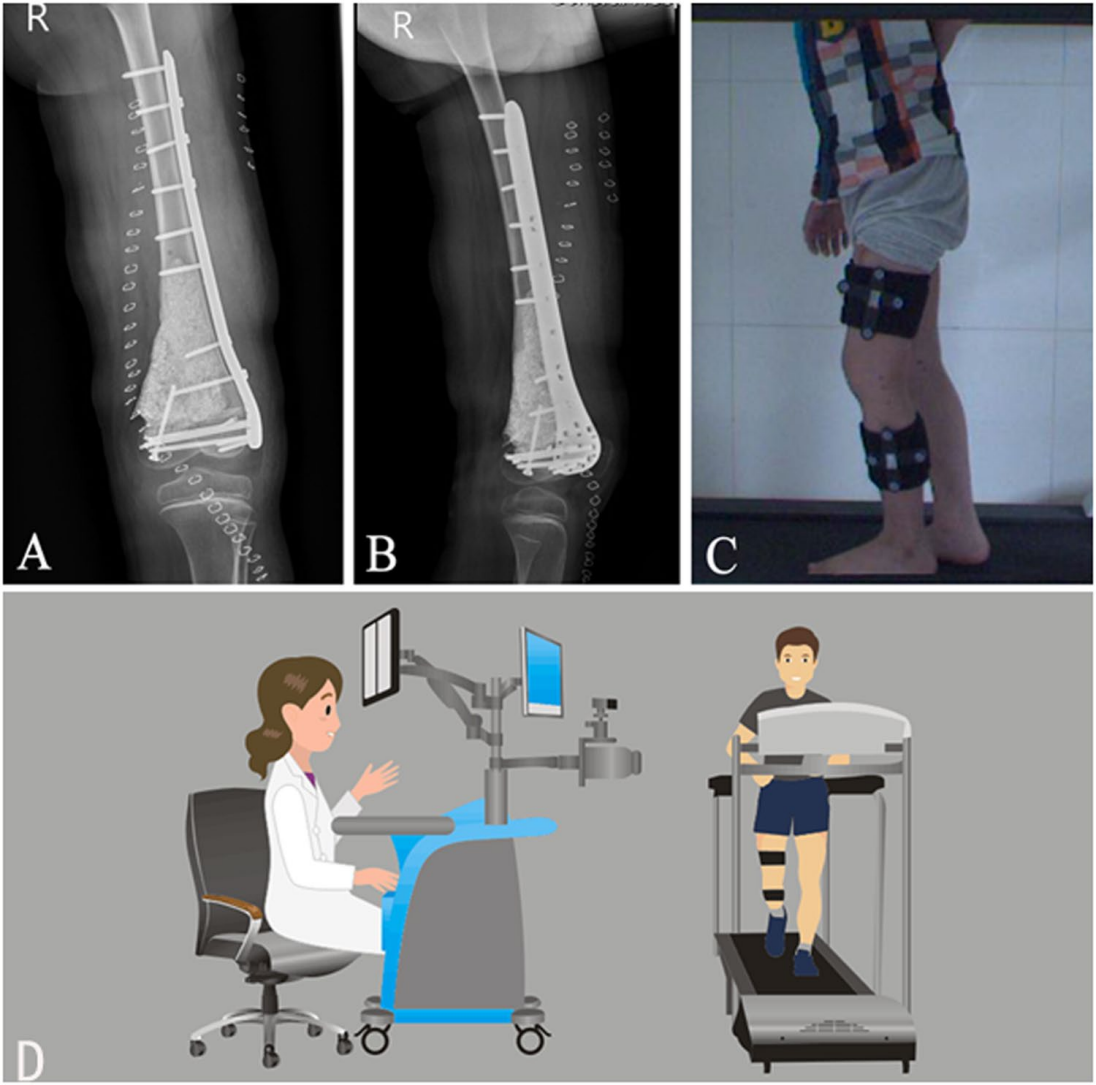
Postoperative radiographs and functional evaluation of knee joint. (**A**,**B**) Postoperative anteroposterior (**A**) and lateral (**B**) radiographs showed that the personalized titanium plate was well-matched to the bone surface. (**C**,**D**) Limb function was evaluated with a novel knee gait analysis system (**C**), which is schematically shown in (**D**). The gait analysis system is equipped with an integrated two-head stereo-infrared camera, infrared light-reflecting markers, a workstation computer with custom software and a treadmill. When the patients were walking on the treadmill, the trajectories of markers attached to the thigh and shank were captured by the stereo-infrared camera, and the tibiofemoral gait analysis was performed in real time.

Postoperative anteroposterior and lateral radiographs showed that the customized plate was well-matched to the bone surface (Fig. 5A,B). Ankle extension, flexion exercises and muscle toning were started on day 1 after the surgery and hip flexion and knee flexion exercises were started 3 weeks later. The patient began to walk with crutches 4 weeks after the surgery. He was able to walk independently on postoperative month 3 and kept tumor-free during the 16-month period of follow-up.

### Functional assessment

In our study, a marker-based knee gait analysis system was used to evaluate the knee function in 6DOF gait. This system is equipped with an integrated two-head stereo-infrared camera, infrared light-reflecting markers, a workstation computer with custom software and a treadmill (Fig. 5D). When the patients were walking on the treadmill, the trajectories of the markers attached to the thigh and shank were captured by the stereo-infrared camera, and the tibial/femoral gait analysis was performed in real time^21^ (Fig. 5C). Functional status of patients’ knees was quantitatively and objectively recorded during the follow-ups (Table 3). The result of ROMs in 6DOF of 12 patients is comparable with the normative data of Chinese knees that was published by us^21^, suggesting the patients have a good status for knee function during their daily activity. One significant contribution to this outcome may be the design of several smaller holes on the distal plates for the ligamentous reconstruction. This allowed for maximum retention of the knee joint due to the mechanical construction. However, it also delayed the timing of postoperative knee flexion exercises. Furthermore, specific scales of the knee function based on this gait analysis system may be needed for future research.

## Discussion

The aim of the current study was to improve the clinical outcomes of microwave ablation for bone tumor around the knee by using the customized plates. The CAD/CAM techniques were utilized for preoperative evaluation, surgical simulation, plate design and manufacturing. A novel knee gait analysis system (Opti_knee) was also introduced to quantitatively evaluate the postoperative knee function. The relatively low risk of the postoperative fractures and the good function of the knee joints were both observed in this study. Additionally, our study indicated that other potential advantages of the customized plates, such as more secure fixation, reduced operation time, and preclusion of plate contouring, over conventional non-personalized plates should be considered in future investigations.

Tumor treatment by microwave-induced hyperthermia *in situ* followed by prophylactic fixation using non-personalized titanium plates for bone tumors of the extremities (176 cases) and pelvis (37 cases) was firstly reported by Fan *et al*.^4^. In their work, excellent clinical outcomes were obtained during the averaged 49-month follow-up. However, they found that pathological fractures after surgery (9 in 130 patients) are an extremely challenging problem. We did not observe the postoperative fractures during the average 29-month follow-ups due to the use of personalized titanium plates for fixation. Furthermore, the customized and site-specific plates were observed to match the bone surfaces well during the surgeries from postoperative radiographies, which was a benefit of the patient-specific design of the plates. The use of the personalized titanium plates avoids the stress concentration in the plates and the resultant implant failures or postoperative fractures. In addition, the results of biomechanical testing described above suggested that the customized plates have superior mechanical properties compared to the conventional plates.

Modern techniques, such as CAD/CAM and RP, have been applied in varies fields of medicine, offering new routes towards the surgery planning and precise osteotomy^15–18, 23–25^. Xu *et al*.^17^ has reported the clinical implementation of custom-made plating using the CAD/CAM technique for acetabular fractures and achieved excellent outcomes. By similar means, in the present study, we used personalized titanium plates for the management of bone tumor. The location of the plates as well as the distribution and length of each screw were all determined before the surgery. In that case, neither more intra-operative depth measuring nor plate contouring was needed. Hence, the time needed for the surgery could be reduced, though no comparison group was included. It should be noted that the operation time and blood loss could vary dramatically due to multiple factors (e.g., patient’s age and general conditions, types and size of tumor, surgical skill, etc)^4, 15, 16^. Several complementary screws were fastened in 4 cases to enhance the fixation because the cavities were found bigger than the preoperative measurements after the curettage. This limitation of the preoperative management may result from the radiological artifact of tumor margin in these cases. No more than 2 screws in each patient were replaced or modulated according to our observations during the surgeries.

We manufactured RP models of tumor-bearing bone segments based on the patients’ CT and MRI data to pretest the attachment of the plates and simulate surgeries. The lesion areas were made in different colors from the surrounding normal tissue (Fig. 1C and D), making the surgery simulations more visualized and precise. The RP models play a critical role in familiarizing both the patients and surgeon’s assistants with the surgical procedure, which was helpful to our clinical practice.

Endoprosthetic replacement is the most popular limb-salvage surgery for the malignant bone tumor around the knee^2, 26–28^. Excellent results regarding the knee function in the early-staged follow-ups have been achieved and reported from a number of studies^25, 29^. The mean MSTS score of the 12 patients reported in present study (27.13) was higher than that reported by Muscolo *et al*., which was obtained from the 36-month follow-up of high-grade metaphyseal osteosarcoma treated with allograft reconstruction^29^, while the value of maximum knee flexion was much closer to what they reported (111.8° vs. 115°). However, compared with the report of the conventional microwave-induced hyperthermia for bone tumor of the extremities^4^, we achieved improved results of knee function due to the use of the personalized plates in this study.

## Conclusion

In conclusion, this study integrated the computer aided design and 3D printing to design a plate that is customized to the anatomical structure of bone involved in the surgical resection of bone tumor. We found that the use of such plate in the bone tumor surgery resulted in improved clinical outcomes. Specifically, the mean Musculoskeletal Tumor Society score as well as mean maximum flexion of affected knees were improved.

## Electronic supplementary material

supporting figures
