# Supplementary material for: 3D printed personalized titanium plates improve clinical outcome in microwave ablation of bone tumors around the knee
Source: Sci Rep. 2017 Aug 8;7:7626. doi: 10.1038/s41598-017-07243-3 (PMC5548746; doi:10.1038/s41598-017-07243-3)
Supplement: Supplementary file 1 — supporting figures [file 41598_2017_7243_MOESM1_ESM.pdf]

Supplementary Materials

3D printed personalized titanium plates improve clinical outcome in microwave ablation of bone tumors around the knee

Limin Ma[†1], Ye Zhou[†1], Ye Zhu,[2] Zefeng Lin[1], Lingling Chen[1], Yu Zhang*[1], Hong Xia*[1] , Chuanbin Mao*[2,3]

1. Department of Orthopedics, Guangdong Key Lab of Orthopedic Technology and Implant, Guangzhou General Hospital of Guangzhou Military Command, 111 Liuhua Road, Guangzhou 510010, China. E-mail: luck_2001@126.com, gzxiahong2@126.com

2. Department of Chemistry and Biochemistry, Stephenson Life Sciences Research Center, University of Oklahoma, Norman OK 73019, USA. E-mail: cbmao@ou.edu

3. School of Materials Science and Engineering, Zhejiang University, Hangzhou, Zhejiang, 310027, China.

[†] These authors contributed equally to this work.

Patient one

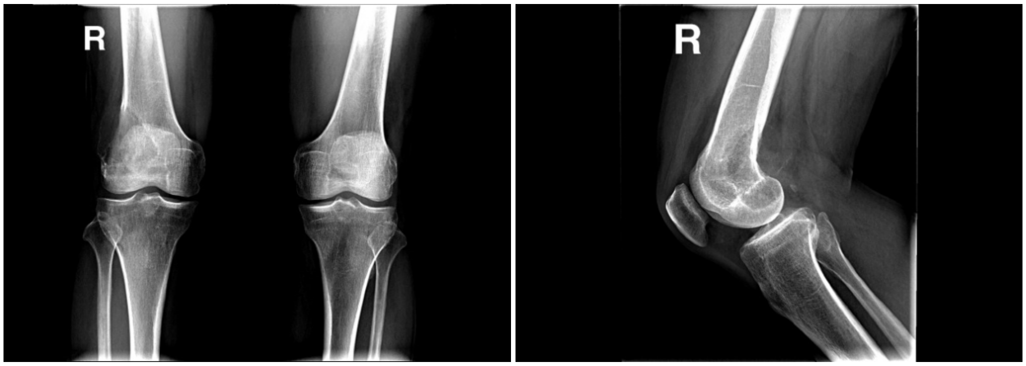

Preoperative radiographs (Left, front view; right, lateral view)

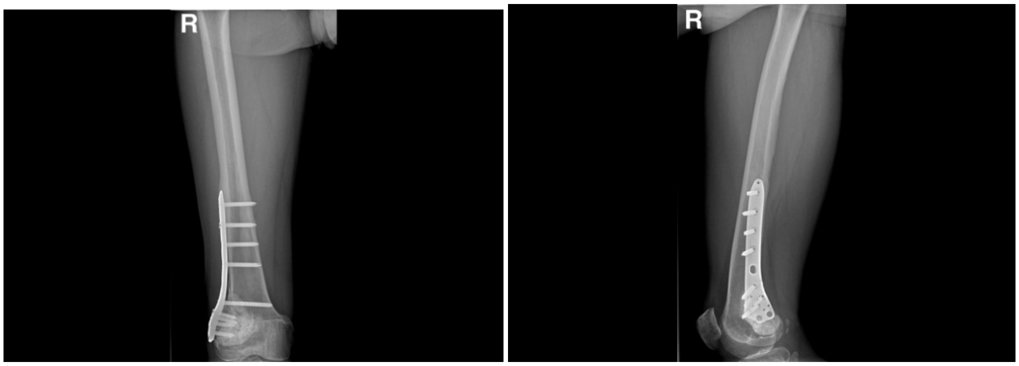

Postoperative radiographs (Left, front view; right, lateral view)

Patient two

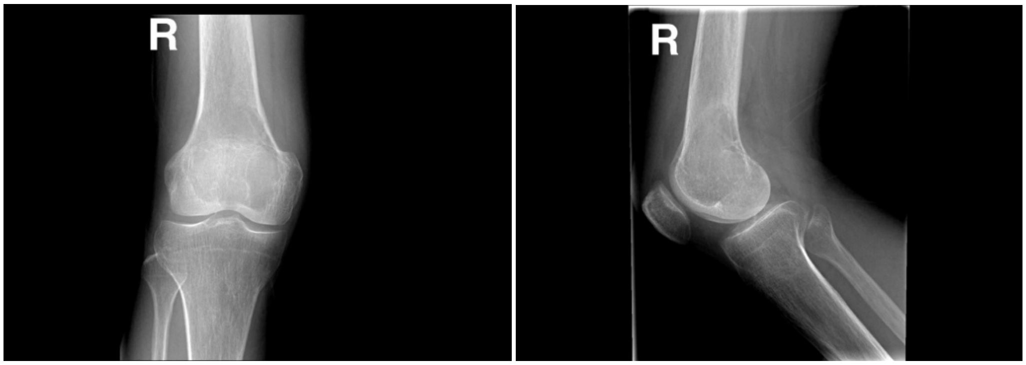

Preoperative radiographs (Left, front view; right, lateral view)

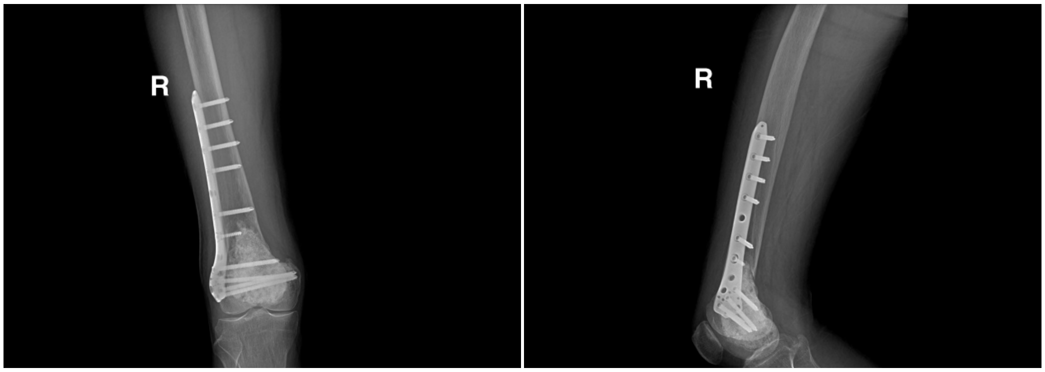

Postoperative radiographs (Left, front view; right, lateral view)

Patient three

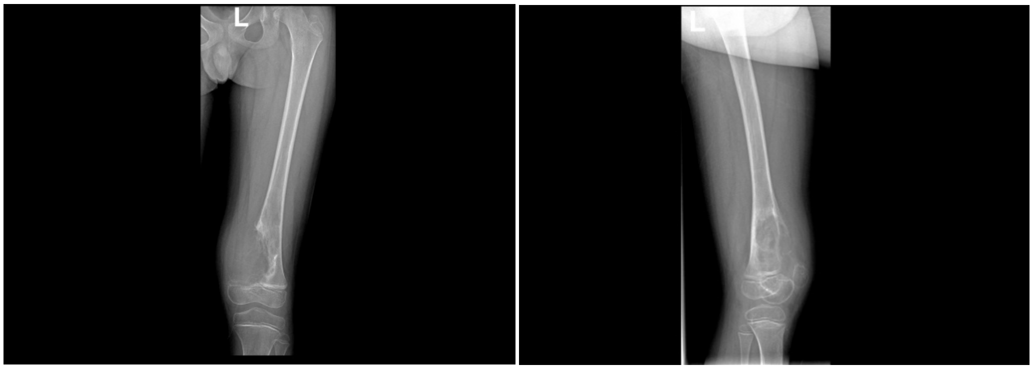

Preoperative radiographs (Left, front view; right, lateral view)

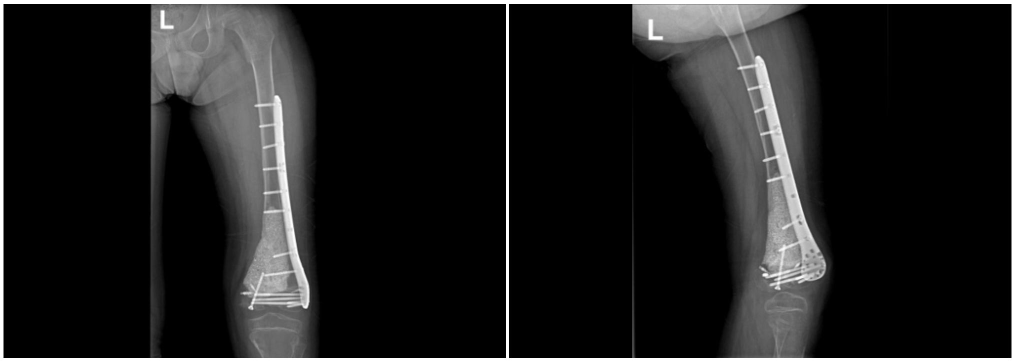

Postoperative radiographs (Left, front view; right, lateral view)

Patient four

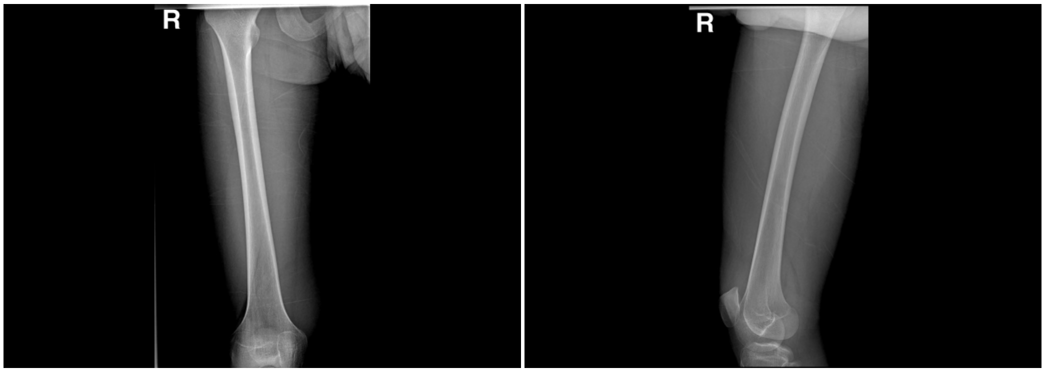

Preoperative radiographs (Left, front view; right, lateral view)

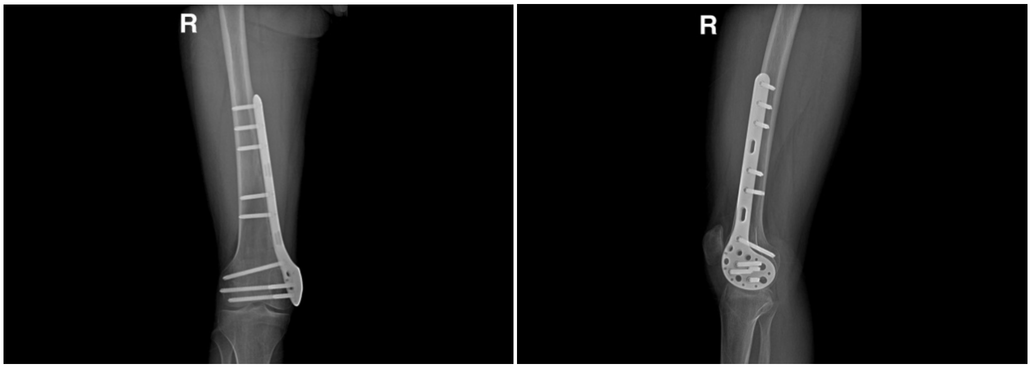

Postoperative radiographs (Left, front view; right, lateral view)

Patient five

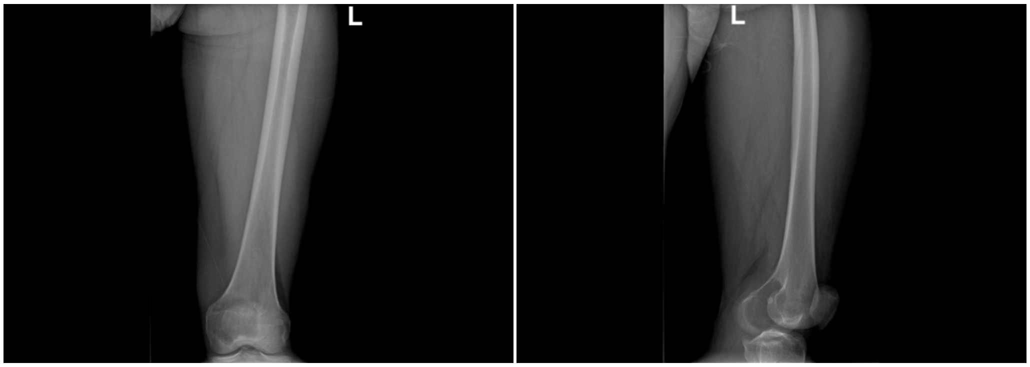

Preoperative radiographs (Left, front view; right, lateral view)

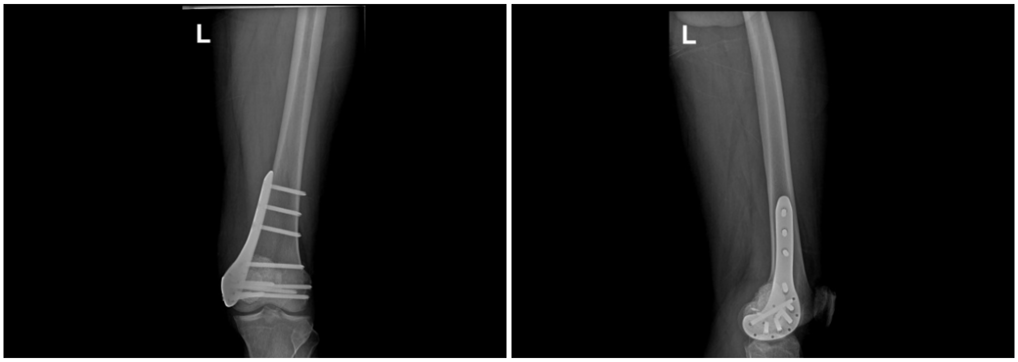

Postoperative radiographs (Left, front view; right, lateral view)

Patient six

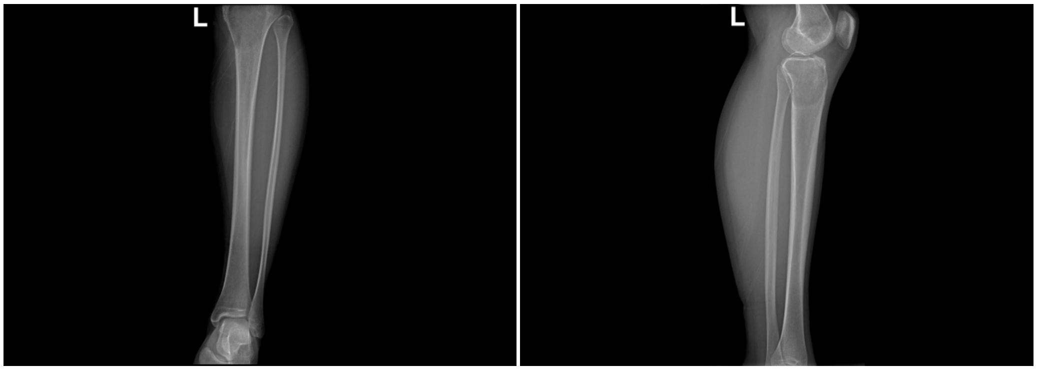

Preoperative radiographs (Left, front view; right, lateral view)

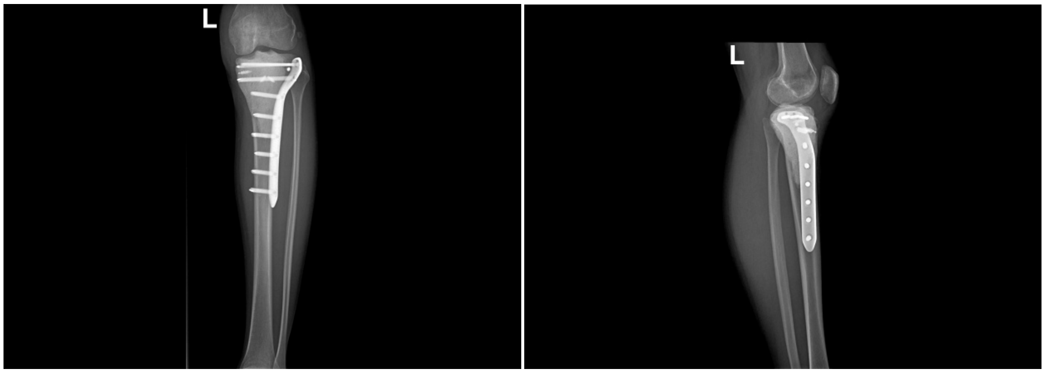

Postoperative radiographs (Left, front view; right, lateral view)

Patient seven

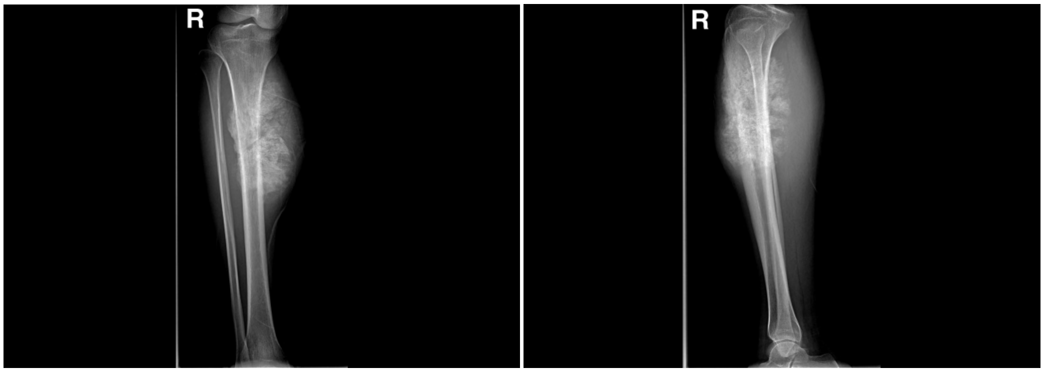

Preoperative radiographs (Left, front view; right, lateral view)

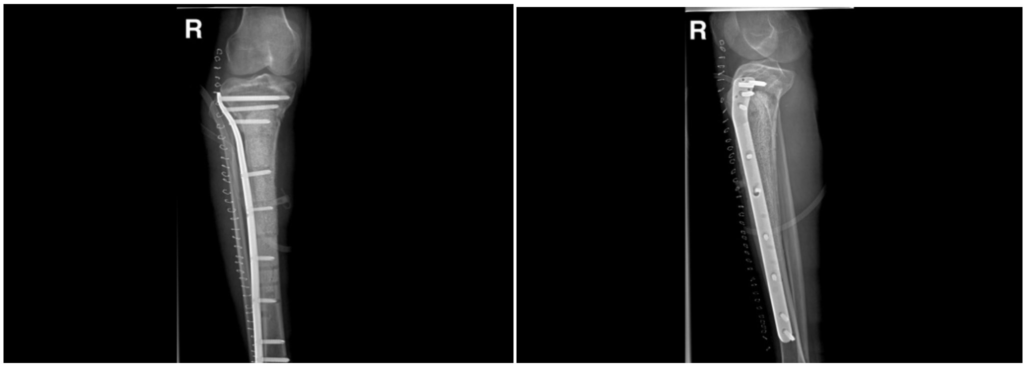

Postoperative radiographs (Left, front view; right, lateral view)

Patient eight

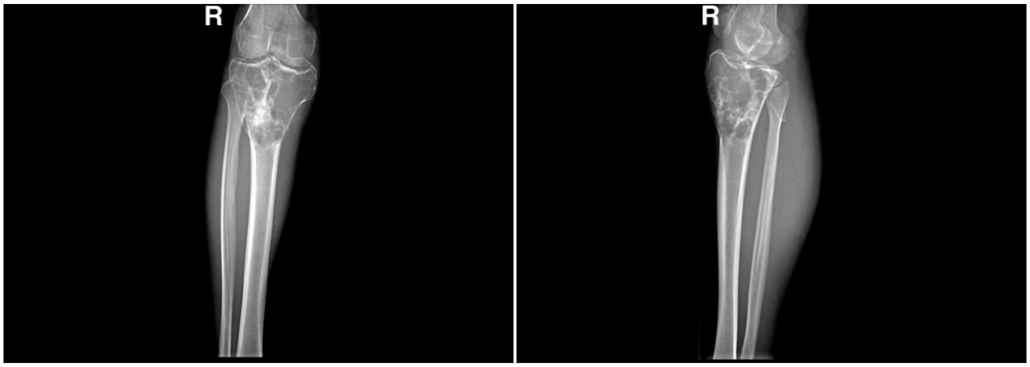

Preoperative radiographs (Left, front view; right, lateral view)

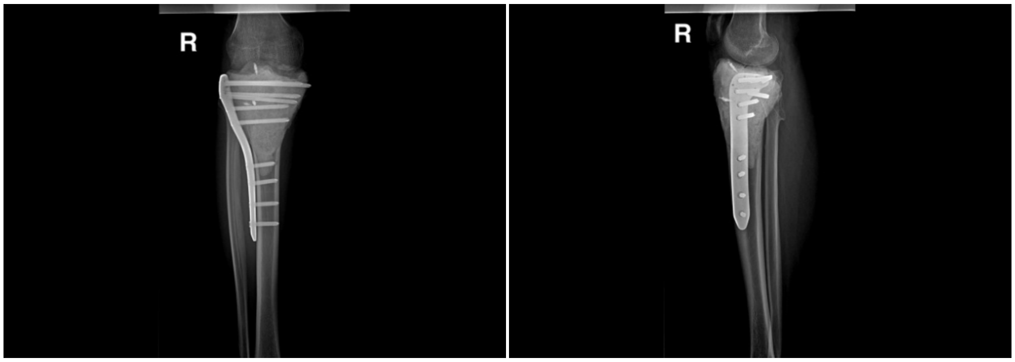

Postoperative radiographs (Left, front view; right, lateral view)

Patient nine

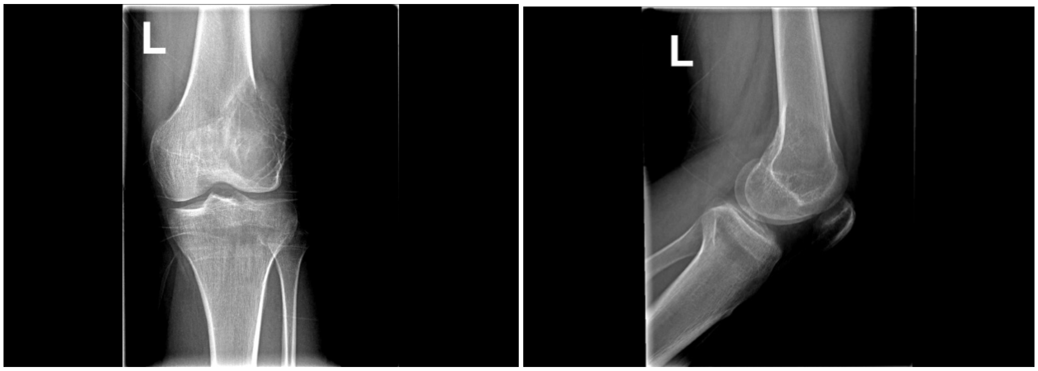

Preoperative radiographs (Left, front view; right, lateral view)

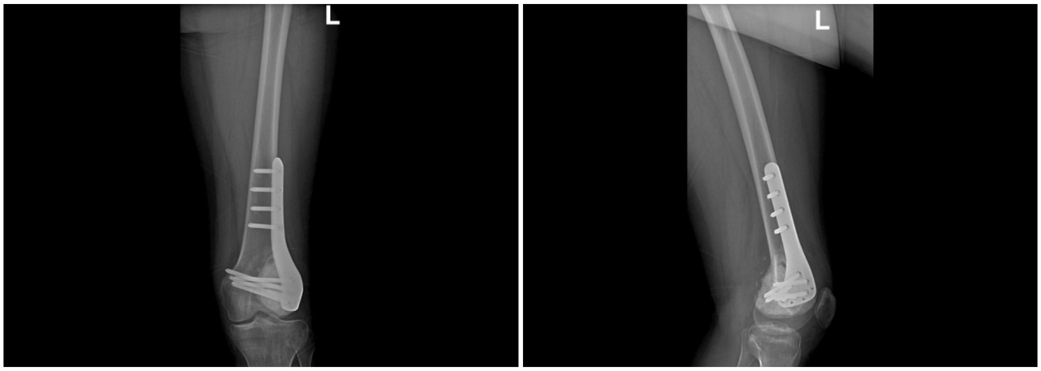

Postoperative radiographs (Left, front view; right, lateral view)

Patient ten

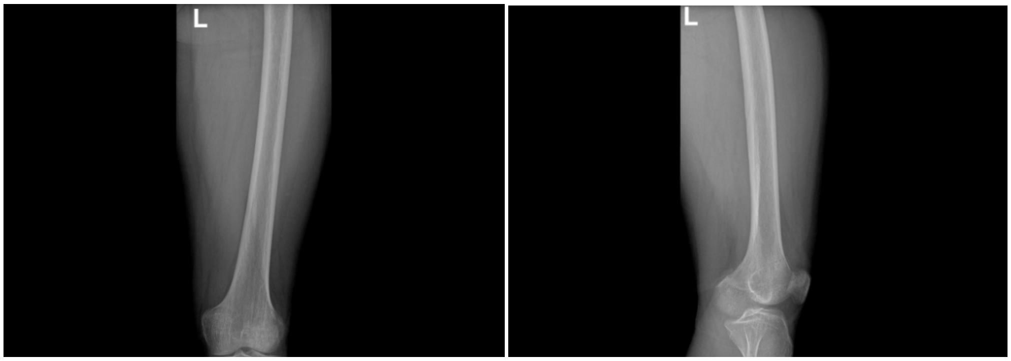

Preoperative radiographs (Left, front view; right, lateral view)

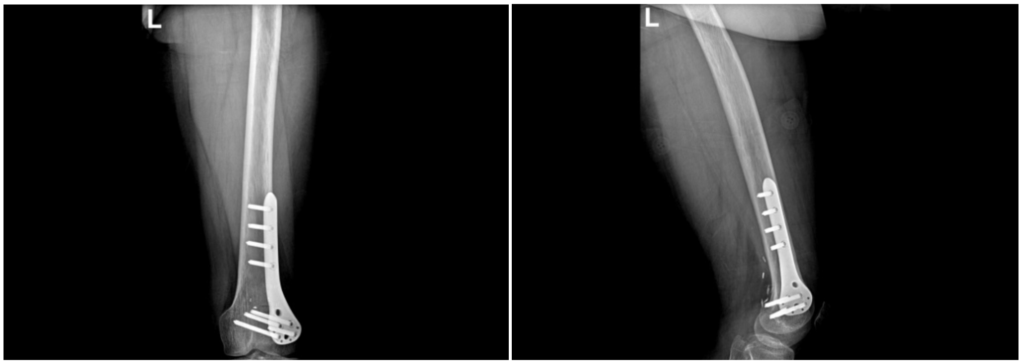

Postoperative radiographs (Left, front view; right, lateral view)

Patient eleven

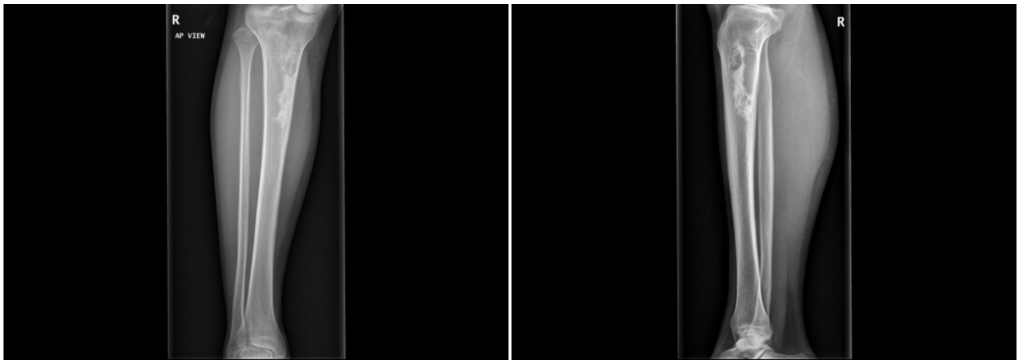

Preoperative radiographs (Left, front view; right, lateral view)

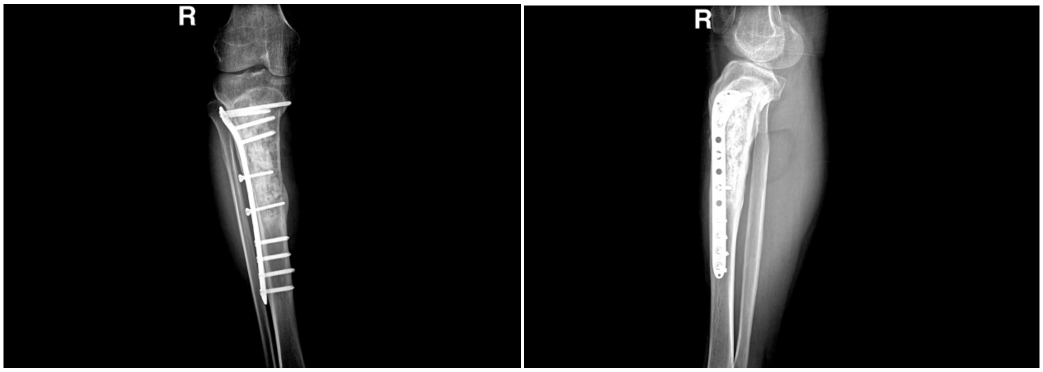

Postoperative radiographs (Left, front view; right, lateral view)

Patient twelve

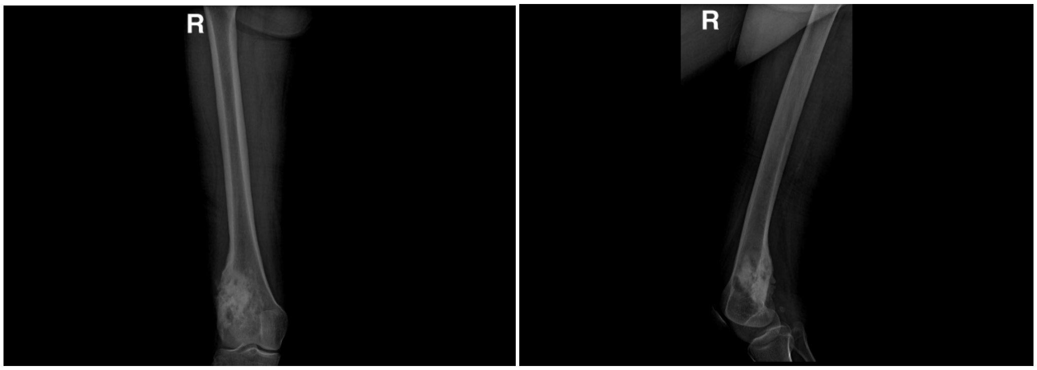

Preoperative radiographs (Left, front view; right, lateral view)

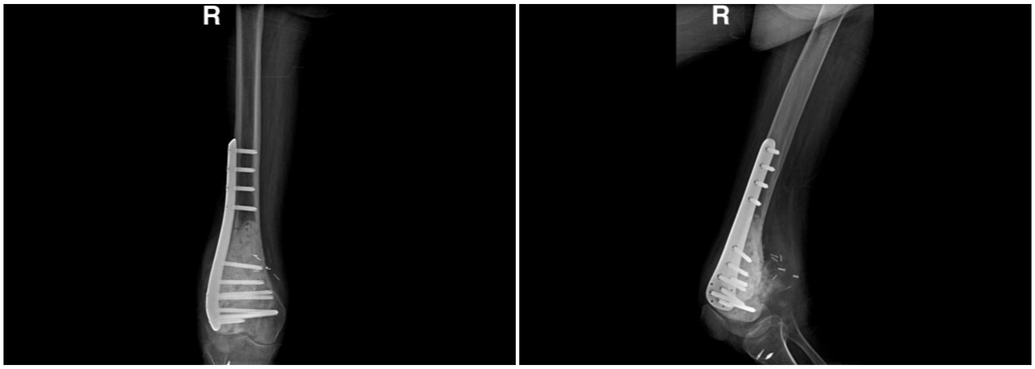

Postoperative radiographs (Left, front view; right, lateral view)
